# Virus-Mediated Targeted DNA Methylation Illuminates the Dynamics of Methylation in an Endogenous Plant Gene

**DOI:** 10.3390/ijms22084125

**Published:** 2021-04-16

**Authors:** Go Atsumi, Kouki Matsuo, Noriho Fukuzawa, Takeshi Matsumura

**Affiliations:** Bioproduction Research Institute, National Institute of Advanced Industrial Science and Technology, Sapporo, Hokkaido 062-8517, Japan; go-atsumi@aist.go.jp (G.A.); matsuo-kouki@aist.go.jp (K.M.); noriho-fukuzawa@aist.go.jp (N.F.)

**Keywords:** DNA methylation, virus vector, RdDM, transcriptional gene silencing

## Abstract

DNA methylation maintains genome stability and regulates gene expression in plants. RNA-directed DNA methylation (RdDM) is critical for appropriate methylation. However, no efficient tools are available for the investigation of the functions of specific DNA methylation. In this study, the cucumber mosaic virus vector was used for targeted DNA methylation. Methylation was rapidly induced but gradually decreased from the 3′ end of the target endogenous sequence in *Nicotiana benthamiana*, suggesting a mechanism to protect against the ectopic introduction of DNA methylation. Increasing 24-nt siRNAs blocked this reduction in methylation by down-regulating *DCL2* and *DCL4*. RdDM relies on the sequence identity between RNA and genomic DNA; however, this identity does not appear to be the sole determinant for efficient DNA methylation. The current findings provide new insight into the regulation of DNA methylation and promote additional effort to develop efficient targeted DNA methylation in plants.

## 1. Introduction

DNA methylation is an important epigenetic mark for regulating genome stability and gene expression in plants. DNA methylation plays a critical role in several biological processes, including the development and response to biotic and abiotic stresses. RNA-directed DNA methylation (RdDM) mediates de novo DNA methylation. The general RdDM model in *Arabidopsis thaliana* [1] suggests that RNA polymerase IV (Pol IV) transcribes RNA (P4-RNA) at target loci. P4-RNAs are converted into double-stranded RNAs (dsRNAs) by RNA-dependent RNA polymerase 2 (RDR2). dsRNAs are cleaved into 24-nt siRNAs by Dicer-like protein 3 (DCL3), and siRNAs are loaded onto ARGONAUTE (AGO) proteins, including AGO4, AGO6, and AGO9. siRNAs/AGOs complexes pair with homologous scaffold RNAs transcribed by Pol V, which is recruited and regulated by DNA methylation reader proteins, including the suppressor of variegation 3–9 homolog protein 2 (SUVH2) and SUVH9, ATPaes, microrchidia 1 (MORC1) and MORC6, and DDR complex containing defective in RNA-directed DNA methylation 1 (DRD1), defective in meristem silencing 3 (DMS3), and RNA-directed DNA methylation 1 (RDM1). Domains rearranged methylase 2 (DRM2) then interacts with AGO4 and methylates DNA at target loci.

DNA methylation for transposon control and gene regulation at specific loci has been investigated in previous studies with mutants that show a global change in DNA methylation. Caution is needed in concluding that any observed change directly reflects the regulation at a target region. One strategy to overcome this problem is targeted DNA methylation/demethylation. Targeted methylation of a foreign gene has been successfully achieved by expressing homologous RNAs with a target sequence from a transgene [2,3,4,5,6]. Targeted methylation of an endogenous gene was reported in petunia [4], maize [7], potato [8], rice [6,9], and *A. thaliana* [10,11], though silencing of the endogenous gene was less efficient as compared to an exogenous gene [6,12]. Zinc-finger and CRISPR technologies used for gene editing were successfully applied to targeted DNA methylation. Johnson et al. demonstrated that Zinc-finger-fused SUVH9 enabled targeted DNA methylation in *A. thaliana FWA* gene [13]. Recent studies also showed that several zinc-finger-fused RdDM components could induce targeted DNA methylation [14] and CRISPR/CAS-based recruitment of tobacco DRM could also achieve targeted DNA methylation in *A. thaliana FWA* gene [15]. For targeted demethylation, the zinc-finger-fused ten-eleven translocation 1 (TET1) catalytic domain could induce DNA demethylation at the *FWA* gene and *CACTA1* transposon in *A. thaliana* [16].

Both expression of homologous RNAs against target regions and utilization of effector proteins for gene editing are powerful tools, and these approaches require transgenic plants that are laborious and time-consuming to develop. Further, transgenic technology is only useful if experimental systems are available to create transgenic plants (e.g., gene delivery into plant cells and regeneration of plants). Plant virus vector technology can overcome these problems since it does not require transgenic plants. Targeted methylation of a foreign gene by a plant virus vector was achieved by expressing RNA identical to the target sequence [17,18,19,20]. Similar methylation of endogenous genes has also been achieved in petunia [21,22], tomato [21], and *A. thaliana* [23]. However, as observed in transgene expression, targeted DNA methylation using a plant virus vector against endogenous genes was less efficient compared to methylation of exogenous genes. Further, success has only been achieved for limited genes in a few plant species. More efficient targeted DNA methylation by plant virus vectors will require a deeper understanding of the characteristics of targeted DNA methylation. At present, such understanding is limited.

In this study, characteristics of targeted DNA methylation against an endogenous gene using a cucumber mosaic virus (CMV) vector in *Nicotiana benthamiana* was investigated. Previous successful studies used virus vectors to target regions that are natively regulated by DNA methylation in tomato *LeSPL-CNR* [21] and *A. thaliana FWA* [23], but this study targeted the promoter region in the phytoene desaturase (*PDS*) gene, which does not appear to be under native regulation by DNA methylation. This strategy was adopted to avoid intrinsic regulation of DNA methylation that would complicate the interpretation of outcomes. The tetraploid plant, *N. benthamiana* [24], was chosen as a model because more than 70% of flowering plants are polyploids and epigenetic control plays a critical role in genome evolution [25]. CMV is a positive-stranded RNA virus consisting of three RNA segments, RNA1-3. CMV infects a variety of plants, including more than 1200 species in over 100 families [26]. A CMV vector based on the CMV-Y strain has been developed for gene silencing in plants, including *N. benthamiana* [20,27], *Glycine max* [28], *Capsicum annuum* [29], and for efficient gene expression in *N. benthamiana* [30,31]. Targeted DNA methylation by the CMV vector was achieved against a foreign gene in *N. benthamiana* [20] and also against endogenous genes in petunia and tomato [21].

In this study, we show that CMV vector-induced DNA methylation in the *PDS* gene’s promoter region is dynamically changed in *N. benthamiana*. The vector induces DNA methylation within two weeks at all cytosine contexts, but the methylation level gradually decreased from the 3′ end of the targeted region. Increasing 24-nt siRNAs instead of 21-nt and 22-nt siRNAs by down-regulating *DCL2* and *DCL4* expression leads to enhanced durability of the high DNA methylation state. Targeted DNA methylation is also induced in the region that contains mismatch sequences to expressed RNAs, suggesting that sequence identity may not be the sole determinant of the efficiency of DNA methylation.

## 2. Results

### 2.1. CMV Vector Induces DNA Methylation Specifically in the Targeted Promoter Sequences of the PDS Gene

**Figure 1:**
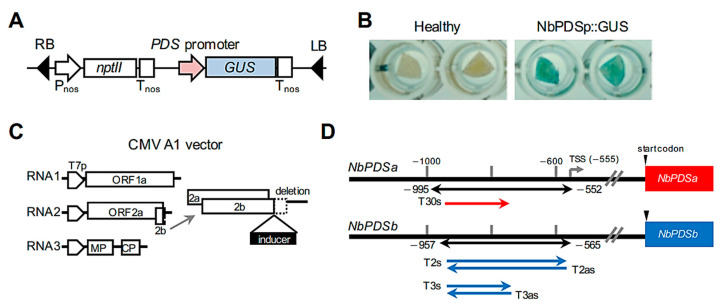
Experimental system for virus-mediated targeted DNA methylation against *PDS* gene in *Nicotiana benthamiana.* (**A**,**B**) Promoter analysis of *PDS* gene in *N. benthmaiana* leaf tissues. Schematic representation of binary plasmid containing the *NbPDSb* promoter (−1225 to −1) -fused GUS gene fragment (NbPDSp::GUS) (**A**). GUS staining of leaf discs collected from leaves where GUS gene was expressed transiently by agrobacterium carrying NbPDSp::GUS (**B**). (**C**) Schematic representation of the cucumber mosaic virus (CMV) A1 vector. T7p = T7 promoter, MP = movement protein, CP = coat protein. (**D**) Inducers for DNA methylation. The red arrow and blue arrows indicate *NbPDSa*-based and *NbPDSb*-based inducers, respectively. The black arrow indicates the region analyzed in bisulfite sequencing. TSS = transcriptional start site [33].

The phytoene desaturase (*PDS*) gene was chosen as a model for characterizing virus-induced targeted DNA methylation. Two copies of the *PDS* gene (designated as *NbPDSa* and *NbPDSb* in this study) are found in *N. benthamiana* draft genome sequences [32]. The region, −1225 to −1 of *NbPDSb*, had promoter activity via transient expression of β-glucuronidase (GUS)-fused construct using agroinfiltration in *N. benthamiana* leaf tissues (Figure 1A,B).

**Figure 2:**
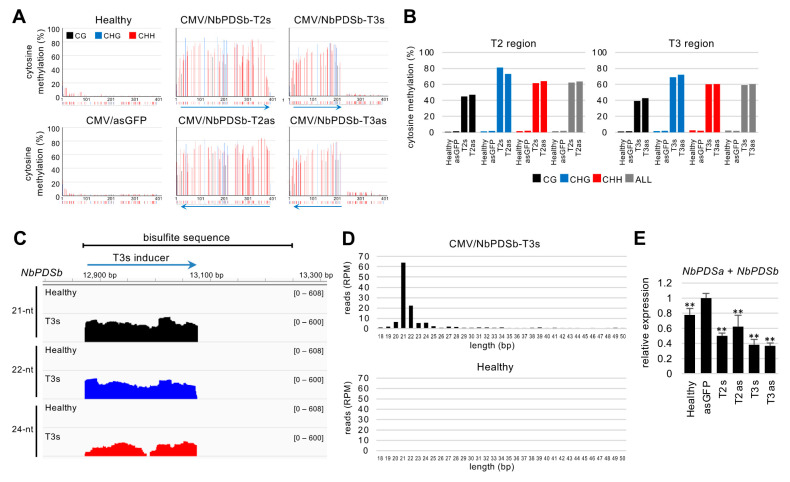
Induction of DNA methylation in the promoter region of the *NbPDS* gene using the CMV vector. (**A**) Targeted bisulfite sequence analysis in the promoter region of *NbPDSb* gene (−957 to −565). Genomic DNAs from the upper non-inoculated leaves at 21 days after inoculation were used for the bisulfite sequencing. The black, blue, and red bars indicate CG, CHG, and CHH methylation, respectively. The color bars below each graph indicate the position of cytosine. The blue arrows below the color bars indicate inducers expressed by the CMV vector. (**B**) The percent of cytosine methylation in each context was calculated in the targeted region. The black, blue, red, and gray bars indicate CG, CHG, CHH, and total methylation, respectively. (**C**) IGV snapshots of 21-nt, 22-nt, and 24-nt small RNAs mapped in the region analyzed by bisulfite sequencing (−957 to −565). Small RNA sequencing used the total RNAs from the leaves where the T3s inducer (−938 to −739) was expressed from the CMV vector at 21 days after inoculation. The *y*-axes indicate raw reads on a log scale, normalized by total mapped reads. (**D**) Length distribution of 18–50-nt small RNAs in the targeted region. (**E**) Total mRNA levels of *NbPDSa* and *NbPDSb* were analyzed in the upper non-inoculated leaves infected with CMV/NbPDSb-T2s, -T2as, -T3s, -T3as, and -asGFP (vector control) at 21 days after inoculation. The expression levels were normalized relative to *NbEF1α*. The error bars indicate the standard deviation of four biological replicates. Statistical analysis used the Dunnett’s method. Data from vector control (asGFP) was used as a control for statistical analysis. **, *p* < 0.01.

The DNA methylation inducers for the promoter region using the CMV vector are provided (Figure 1C,D) [20,21]. The bisulfite sequencing indicated that the methylation level around the target region (−957 to −565) was quite low in both plants without CMV infection and plants inoculated with vector control expressing a partial sequence of GFP at 21 days after inoculation (dpi) (Figure 2A). The expression of the T2s inducer RNA significantly induced methylation at all cytosine contexts (CG, CHG, and CHH) throughout the target region (Figure 2A). The average levels of CG, CHG, and CHH methylation in the target region were approximately 45%, 81%, and 62%, and no clear differences in DNA methylation levels were observed between constructs designed in a coding strand (T2s) and an opposite strand (T2as) (Figure 2A,B). When T3s inducer (5′ half of the analyzed area) was expressed, DNA methylation was specifically induced in the targeted region (Figure 2A), and there were no clear differences in DNA methylation levels between constructs based on a coding (T3s) and an opposite strand (T3as) (Figure 2A,B).

Twenty-one-nt, 22-nt, and 24-nt small RNAs play an important role in RdDM. Almost no small RNAs (18–50 nt) accumulated around the region (−957 to −565) in healthy plants (Figure 2C,D). In contrast, various sizes of small RNAs, including 21-, 22-, and 24-nt, accumulated in the targeted region in T3s-expressing plants at 21 dpi (Figure 2C,D). The order of accumulation levels was 21-nt > 22-nt > 24-nt. Secondary siRNAs appeared not to be generated in both upstream and downstream regions (Figure 2C), which is consistent with no spread of DNA methylation from the target region to the downstream region (Figure 2A). mRNA levels of the *NbPDS* gene by real-time PCR show that total mRNA levels of *NbPDSa* and *NbPDSb* decreased in the presence of inducers, and no clear difference between constructs was seen based on coding and an opposite strand (Figure 2E).

### 2.2. Gradual Reduction of DNA Methylation from the 3′ End of the Targeted Region

**Figure 3:**
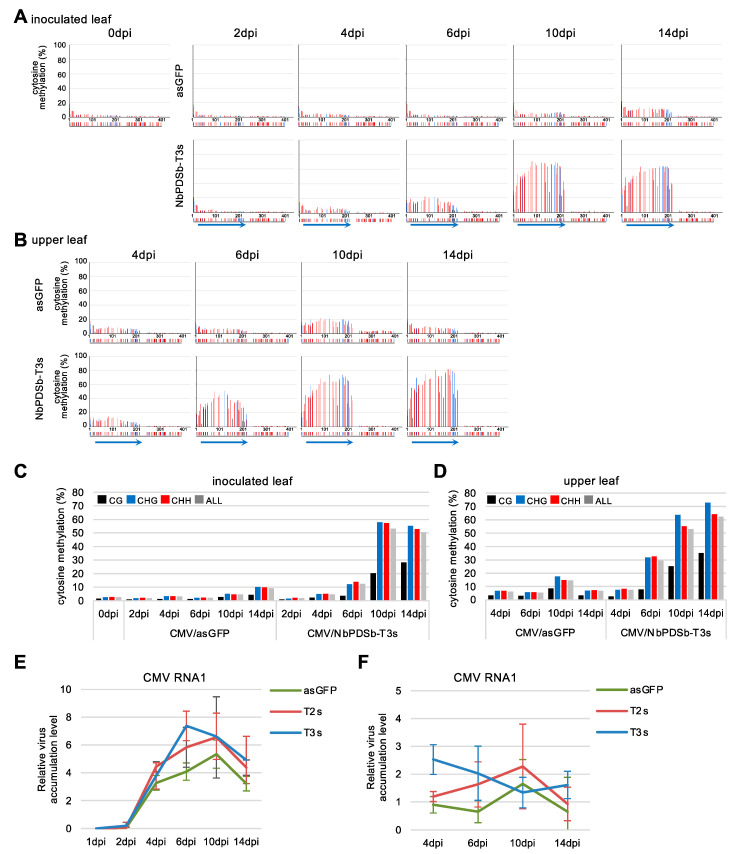
Time-course analysis of DNA methylation induced by the CMV vector in the promoter region of the *NbPDS* gene. (**A**,**B**) Targeted bisulfite sequence analysis in the promoter region of *NbPDSb* gene (−957 to −565). Genomic DNAs from the inoculated leaves at 0, 2, 4, 6, 10, and 14 days after inoculation (**A**) and from the upper non-inoculated leaves at 4, 6, 10, and 14 days after inoculation (**B**) were used for bisulfite sequencing. The black, blue, and red bars indicate CG, CHG, and CHH methylation, respectively. The color bars below each graph indicate the position of cytosine. The blue arrows below the color bars indicate inducers expressed by the CMV vector. (**C**,**D**) The percent of cytosine methylation in the targeted region was calculated in inoculated (**C**) and upper non-inoculated leaf (**D**). The black, blue, red, and gray bars indicate CG, CHG, CHH, and total methylation, respectively. (**E**,**F**) Accumulation level of CMV RNA1 in inoculated (**E**) and upper non-inoculated (**F**) leaves. The accumulation level of viral RNA was normalized relative to that of *NbEF1α*. The error bars indicate the standard deviation of four biological replicates.

**Figure 4:**
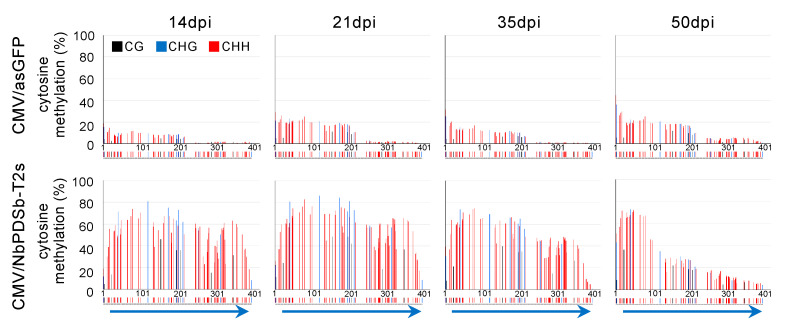
The gradual decline of the DNA methylation level after 21 days post-inoculation. Targeted bisulfite sequence analysis in the promoter region of *NbPDSb* gene (−957 to −565). Genomic DNAs from upper non-inoculated leaves of wild-type plants infected with CMV/NbPDSb-T2s at 14, 21, 35, and 50 days after inoculation were used for bisulfite sequencing. The black, blue, and red bars indicate CG, CHG, and CHH methylation, respectively. The color bars below each graph indicate the position of cytosine. The blue arrows below color bars indicate inducers of DNA methylation.

A time-course analysis of DNA methylation using bisulfite sequencing indicated that DNA methylation in all cytosine contexts began to be induced from 6 dpi in both inoculated (Figure 3A,C) and upper non-inoculated leaves (Figure 3B,D) infected with CMV carrying T3s inducer. This timing corresponds to the time when the virus was significantly accumulated (Figure 3E,F). No spread of DNA methylation to the downstream region was observed at any time points in both inoculated and upper non-inoculated leaves. Unexpectedly, we found that DNA methylation level gradually decreased in all cytosine contexts, especially at the 3′ region of the target region in the *NbPDSb* promoter in plants inoculated with CMV carrying T2s inducer (Figure 4).

**Figure 5:**
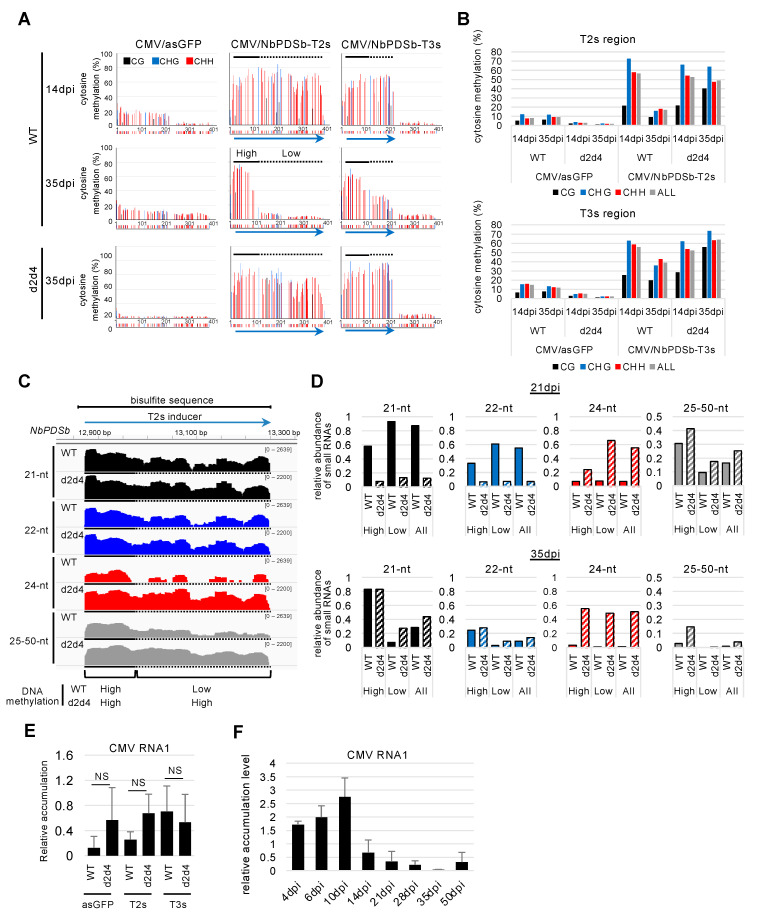
Maintenance of DNA methylation at 35 days after inoculation. (**A**) Targeted bisulfite sequence analysis in the promoter region of *NbPDSb* gene (−957 to −565). Genomic DNAs from upper non-inoculated leaves of wild-type and d2d4 plants at 35 days post-inoculation (dpi) were used for bisulfite sequencing. The black, blue, and red bars indicate CG, CHG, and CHH methylation, respectively. The color bars below each graph indicate the position of cytosine. The blue arrows below the color bars indicate inducers for DNA methylation. Black solid and broken lines indicate high- and low-methylation regions in wild-type relative to d2d4 plants at 35dpi, respectively. (**B**) The percent of cytosine methylation in each context was calculated in the targeted region of *NbPDSb*. The black, blue, red, and gray bars indicate CG, CHG, CHH, and total methylation, respectively. (**C**) Small RNA sequencing used total RNAs from leaves, where the T2s inducer (−938 to −568) was expressed from the CMV vector at 35 days after inoculation. IGV snapshots of 21-nt, 22-nt, 24-nt, and 20–50-nt small RNAs in the region analyzed by bisulfite sequencing (−957 to −565). The *y*-axes indicate raw reads on a log scale, normalized by total mapped reads. Black solid and broken lines indicate high- and low-methylation regions, respectively, as shown in (**A**). (**D**) Reads of 21-nt, 22-nt, 24-nt, and 20–50-nt mapped in T2s-targeted regions were counted in wild-type and d2d4 plants at 21 dpi (data shown in Supplementary Figure S1A) and 35 dpi (**C**). A relative abundance of small RNAs indicates reads per total mapped million reads normalized by region length. “High” indicates the relative abundance of small RNAs mapped to high methylation regions in wild-type plants at 35 dpi (solid black lines in (**A**)). “Low” indicates the relative abundance of small RNAs mapped to low methylation regions in wild-type plants at 35 dpi (broken black lines in (**A**)). (**E**) Virus accumulation level at 35 days after inoculation in the samples used in (**A**–**D**). Accumulation levels measured by real-time PCR were normalized relative to *NbEF1α*. The error bars indicate the standard deviation of three biological replicates. Statistical analyses were conducted using Welch’s *t*-test. NS: not significant. (**F**) Virus accumulation level in upper non-inoculated leaves of wild-type plants infected with CMV/NbPDSb-T2s. The accumulation levels measured by real-time PCR were normalized relative to that of *NbEF1α*. The error bars indicate the standard deviation of four biological replicates.

**Figure 6:**
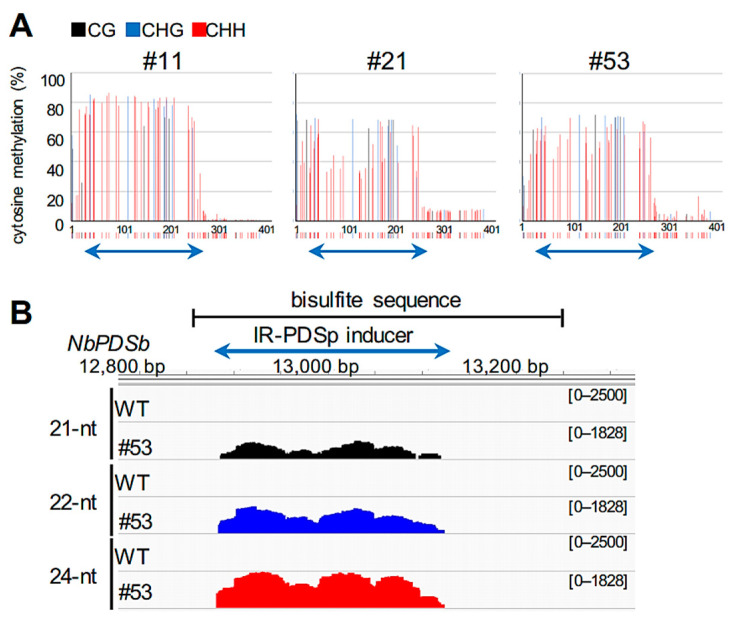
DNA methylation and small RNA profiles in transgenic plants constitutively expressing an inverted repeat sequence of the *NbPDSb* promoter. (**A**) Targeted bisulfite sequence analysis in the promoter region of the *NbPDSb* gene (−957 to −565). Genomic DNA was extracted from the leaves of each transgenic line (line#11, #21, and #53) that carry a single copy of the inverted repeat sequence of the *NbPDSb* promoter (−933 to −681, IR-PDSp). The black, blue, and red bars indicate CG, CHG, and CHH methylation, respectively. The color bars below each graph indicate the position of cytosine. The blue arrows below the color bars indicate the inverted repeat inducers expressed from the transgene. (**B**) Small RNA sequencing used total RNAs from leaves of wild-type and line #53 plants. IGV snapshots of 21-nt, 22-nt, and 24-nt small RNAs in the region analyzed by bisulfite sequencing (**A**). The *y*-axes indicated raw reads on a log scale and were normalized by total mapped reads.

As 24-nt siRNAs are important for maintaining DNA methylation [23], increasing the amount of 24-nt siRNAs was expected to maintain the elevated DNA methylation levels in our system. We used *DCL2* and *DCL4*-double knock-down (d2d4) *N. benthamiana* transgenic plants [34], where increases in 24-nt siRNAs are expected by reducing the transcription of 21-nt and 22-nt siRNAs that are produced by DCL4 and DCL2, respectively [35]. To confirm the increase in 24-nt siRNAs generated from inducers expressed from CMV vector in d2d4 plants, we analyzed the pattern of small RNAs produced from the T2s inducer by small RNA sequencing. The results in 21 dpi showed that most siRNAs produced from the T2s inducer in d2d4 plants were 24-nt siRNAs, which accounted for about 74.3% of total 21-nt, 22-nt, and 24-nt siRNAs, in contrast to about 4.9% in wild-type plants (Supplementary Figure S1). CMV/NbPDSb-T2s and CMV/NbPDSb-T3s were inoculated onto wild-type and d2d4 plants, and methylation levels were assessed. At 35dpi, DNA methylation level at *NbPDSb* was dramatically reduced in wild-type plants but remained high in d2d4 plants (Figure 5A,B). Small RNA sequencing indicated that amounts of 24-nt siRNAs dramatically increased in d2d4 plants in the low-methylation region observed in wild-type plants at 35 dpi (Figure 5C,D; Supplementary Figure S2). Virus accumulation in d2d4 plants is not consistently higher compared to wild-type plants at 35 dpi (Figure 5E). These results suggest that low accumulation of 24-nt siRNAs leads to a reduction in DNA methylation levels in wild-type plants, and enhanced accumulation of 24-nt siRNAs suppresses this reduction in d2d4 plants. Virus accumulation pattern showed that the virus level reached a peak at 10 dpi and declined thereafter (Figure 5F), suggesting that amounts of 24-nt siRNAs generated from inducers decline in a similar fashion, which leads to a reduction of DNA methylation. Consistent with this notion, elimination of DNA methylation was not observed throughout the region in three transgenic plants (T0 generation) that constitutively express an inverted repeat sequence of the NbPDSb promoter along with the accumulation of 24-nt siRNAs (Figure 6). Notably, the amount of 25–50-nt small RNAs, the size of P4RNA suggested to be important for RdDM [36], increased in low methylation regions in d2d4 plants (Figure 5C; Supplementary Figure S2). This observation suggests that increased 25–50-nt small RNAs enhance de novo methylation or accumulate as precursors for 24-nt RNAs.

### 2.3. Sequence Identity May Not Be the Sole Determinant of Efficiency of Silencing and Induction of DNA Methylation of PDS Genes

RdDM induction is homology-dependent, and sequence identity between inducer and target is a critical factor in determining the induction efficiency of DNA methylation. We investigated whether sequence identity affects the induction of DNA methylation and silencing the target genes using the closely related two PDS genes (NbPDSa and NbPDSb) found in the *N. benthamiana* genome. Real-time PCR analysis indicated that basal expression levels of *NbPDSa* were about 4.3× higher than the expression of *NbPDSb* (Supplementary Figure S3).

**Figure 7:**
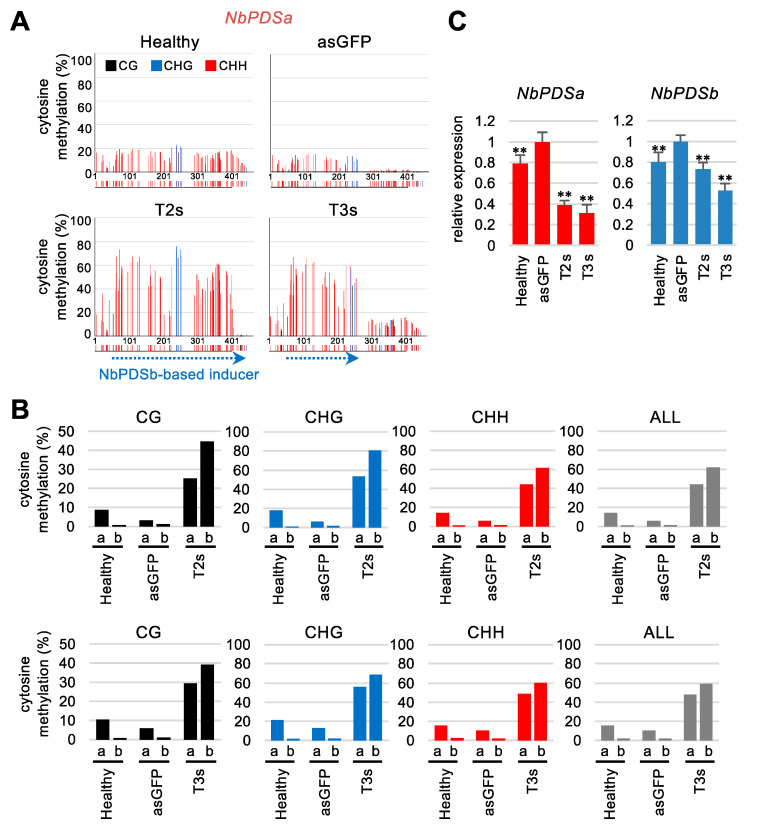
Induction of DNA methylation in the homologous region of the *NbPDSa* gene using a CMV vector. (**A**) Targeted bisulfite sequence analysis in the promoter region of the *NbPDSa* gene (−995 to −552). Genomic DNAs from the upper non-inoculated leaves at 21 days after inoculation were used for bisulfite sequencing. The black, blue, and red bars indicate CG, CHG, and CHH methylation, respectively. The color bars below each graph indicate the position of cytosine. The dashed blue arrows below the color bars indicate regions homologous with inducers expressed by the CMV vector. (**B**) The percent of cytosine methylation in each context was calculated in the targeted region of *NbPDSa* and *NbPDSb* (the same data shown in Figure 2B). The black, blue, red, and gray bars indicate CG, CHG, CHH, and total methylation, respectively. (**C**) mRNA levels of *NbPDSa* and *NbPDSb* were separately quantified by real-time PCR in the upper non-inoculated leaves with CMV/NbPDSb-T2s, -T3s, -asGFP (vector control) at 21 days after inoculation. The expression level of vector control was set to 1.0 for *NbPDSa* and *NbPDSb*. The expression levels were normalized relative to *NbEF1α*. The error bars indicate the standard deviation of four biological replicates. Statistical analyses used the Dunnett’s method. Data from vector control (asGFP) was used as a control for statistical analysis. **, *p* < 0.01.

NbPDSb-based inducers were used in the above experiments, and each inducer has mismatch sequences with *NbPDSa*: T2s and T3s have 84.6% and 87.2% nucleotide identities with corresponding regions of *NbPDSa*, respectively (Supplementary Figure S4). We found that both T2s and T3s efficiently induced DNA methylation in the homologous region of *NbPDSa* (Figure 7A,B). Further, the DNA methylation level of *NbPDSa* was lower than that of *NbPDSb* in all cytosine contexts (Figure 7A,B). Unexpectedly, regardless of the lower DNA methylation level in *NbPDSa*, both *NbPDSb*-based T2s and T3s reduce *NbPDSa* expression more efficiently compared to *NbPDSb* expression (Figure 7C). Also, the time-course analysis indicated that *NbPDSa* mRNA was constantly reduced compared to *NbPDSb* mRNA in both inoculated (Supplementary Figure S5A) and upper non-inoculated leaves (Supplementary Figure S5B). These results indicated that NbPDSb-based inducer reduced *NbPDSa* mRNA more efficiently than *NbPDSb* mRNA independent of DNA methylation level.

**Figure 8:**
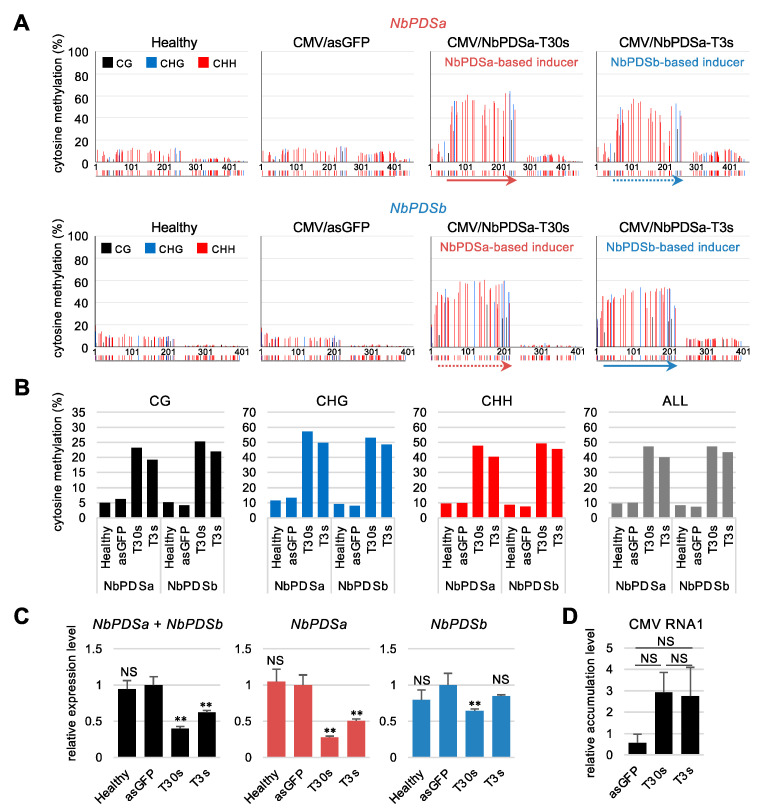
Comparison of DNA methylation and mRNA reduction between *NbPDSa*-based and *NbPDSb*-based inducers. (**A**) Targeted bisulfite sequence analysis in the promoter region of *NbPDSa* gene (−995 to −552) and *NbPDSb* gene (−957 to −565). Genomic DNAs from the upper non-inoculated leaves at 12 days after inoculation were used for bisulfite sequencing. The black, blue, and red bars indicate CG, CHG, and CHH methylation, respectively. The color bars below each graph indicate the position of cytosine. Red and blue arrows below the color bars indicate *NbPDSa*-based and *NbPDSb*-based inducers, respectively. The dashed arrows indicate regions homologous with inducers. (**B**) The percent of cytosine methylation in each context was calculated in the targeted region of *NbPDSa* and *NbPDSb*. The black, blue, red, and gray bars indicate CG, CHG, CHH, and total methylation, respectively. (**C**) mRNA levels of *NbPDSa* and *NbPDSb* were separately quantified in the upper non-inoculated leaves with CMV/NbPDSb-T2s, -T3s, -asGFP (vector control) at 12 days after inoculation. The expression level of vector control was set to 1.0. The expression levels were normalized relative to that of *NbEF1α*. The error bars indicate the standard deviation of three biological replicates. Statistical analyses were conducted using the Dunnett’s method. Data from vector control (asGFP) was used as a control for statistical analysis. **, *p* < 0.01; NS: not significant. (**D**) CMV accumulations in upper non-inoculated leaves at 12 days after inoculation. Virus accumulation levels were normalized relative to *NbEF1α*. The error bars indicate the standard deviation of three biological replicates. Statistical analyses were conducted using the Tukey–Kramer method. NS, not significant.

The reciprocal experiment for inducing DNA methylation by the NbPDSa-based inducer (T30s) revealed, by bisulfite sequencing, that NbPDSa-based T30s efficiently induced DNA methylation against the *NbPDSb* sequence (Figure 8A,B). The level of DNA methylation in *NbPDSb* sequences induced by the NbPDSa-based T30s was higher than that induced by the NbPDSb-based T3s in all cytosine contexts. Further, T30s’ expression reduced both *NbPDSa* and *NbPDSb* mRNA levels more effectively compared to T3s (Figure 8C), though no significant difference in virus accumulation was seen (Figure 8D). NbPDSa-based T30s is an effective DNA methylation inducer for *NbPDSb* regardless of the presence of mismatches, suggesting that sequence identity alone does not determine the efficiency of induction of DNA methylation.

## 3. Discussion

Virus-mediated targeted DNA methylation has significant advantages over transgenic technology, but is to date, poorly characterized. In this work, we reveal rapid induction and gradual reduction of DNA methylation in the promoter sequence of the *PDS* gene using a CMV vector in *N. benthamiana*. To the best of our knowledge, this report is the first to capture dynamic changes in DNA methylation in an endogenous genomic region in plants.

Previous work showed that DNA methylation is not induced in the targeted region of the *FWA* gene in *A. thaliana* infected with a TRV-carrying target fragment of *FWA* [23]. The lack of DNA methylation in the *FWA* gene might be due to intrinsic epigenetic regulation of the *FWA* gene in *A. thaliana*. The *PDS* gene in *N. benthamiana* is apparently not regulated by DNA methylation, and DNA methylation in this gene would not be influenced by intrinsic regulation. Even so, T2s-induced DNA methylation gradually declined (Figure 4). Small RNA sequencing showed a positive correlation between levels of 24-nt siRNA and DNA methylation. A model from a previous study may provide an explanation [23]. Where initiation and establishment of RdDM are mediated by 21-nt and 22-nt siRNAs, and induced DNA methylation is reinforced and maintained by 24-nt siRNAs: (1) inducer dsRNA generated from the CMV vector are cleaved into 21-nt and 22-nt siRNAs by DCL4 and DCL2, respectively (small amounts of 24-nt siRNAs are also generated by DCL3); (2) 21/22-nt siRNAs are incorporated into ARGONAUTE proteins, and cytosine is methylated via the Pol V pathway; (3) once methylation is established, canonical Pol IV-mediated RdDM machinery is recruited to a target region, and cytosine methylation is reinforced and maintained through 24-nt siRNAs. At 35 dpi in the 3′ low methylation region of wild-type plants (Figure 5A,B), production of 21/22-nt and 24-nt siRNAs was low due to reduced virus accumulation for efficient initiation/establishment and reinforcement/maintenance of DNA methylation, respectively (Figure 5C,D). In d2d4 plants, high levels of 24-nt siRNAs might maintain DNA methylation (Figure 5C,D). Notably, 25–50-nt small RNAs may be involved in maintaining DNA methylation in d2d4 plants. Previous reports show that Pol IV generates 25–50-nt small RNAs called P4RNA in *A. thaliana* [36,37,38,39]. Yang et al. found that DNA methylation at many loci do not require DCLs and proposed that P4RNA itself is a trigger for DNA methylation at the initiation phase. P4RNA may also function in establishment/maintenance, independent of 24-nt siRNAs [36]. As the amount of 25–50-nt RNAs was also positively correlated with the level of DNA methylation (Figure 5C), these classes of RNAs might also contribute to the maintenance of high methylation in d2d4 plants. The 25–50-nt RNA might provide evidence that canonical RdDM is newly initiated after the DNA is methylated by a virus-mediated Pol IV-independent non-canonical RdDM pathway.

**Figure 9:**
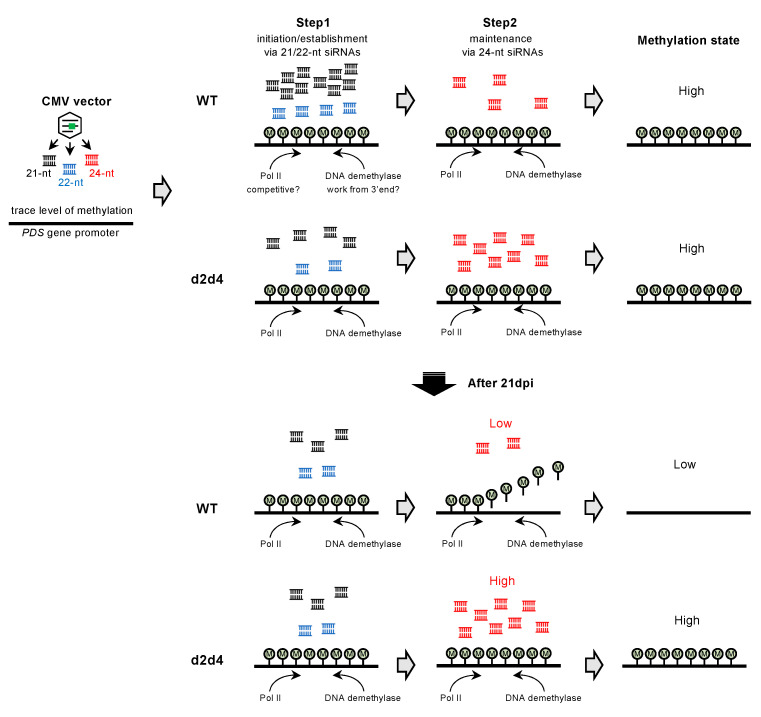
Model for induction and reduction of DNA methylation by the CMV vector in the *N. benthamiana* PDS gene. The CMV vector produces dsRNAs against a target sequence, which are processed into 21-nt, 22-nt, and 24-nt siRNAs by DCL4, DCL2, and DCL3 proteins, respectively. At the early stages of infection (until 21dpi) in wild-type plants, DNA methylation is initiated and established through highly accumulated 21-/22-siRNAs (step1) and maintained via 24-nt siRNAs (step2). In d2d4 plants, though the level of 21-nt and 22-nt siRNAs are low, highly accumulated 24-nt siRNAs effectively maintain high DNA methylation states. At later stages of infection (after 21dpi) in wild-type plants, the introduced DNA methylation is gradually eliminated, presumably due to the reduction in accumulated small RNAs. In contrast, in d2d4 plants, highly accumulated 24-nt siRNAs maintain a high methylation state. The reduction of DNA methylation may be caused by competition between RdDM machinery and polymerase II transcription. Alternatively, but not exclusively, DNA demethylase may be preferentially recruited to the 3′ end of the target region by an unknown mechanism and erase DNA methylation because the DNA methylation appears to be erased from the 3′ end.

The reduction of DNA methylation was biased at the 3′ end of the targeted region of the *PDS* promoter sequence (Figure 4 and Figure 5A). In the *A. thaliana FWA* gene, DNA methylation was introduced in progenies from plants infected with TRV, but it was not always introduced throughout the target region. Some plants showed methylation only at the 5′ end region near the transcription start site [33]. A recent study demonstrates that artificial site-specific transcriptional activation reduces promoter methylation of the *FWA* gene in *A. thaliana* [15]. These reports suggest that transcription by polymerase II perturbs the introduction of DNA methylation near the transcriptional start site (Figure 9). This suggestion is not inconsistent with genome-wide analyses that show low DNA methylation levels in this region [40]. RNA-seq data available in the Sol Genomics Network database [32] indicate an accumulation of the *PDS* gene transcripts with a 5′ end within T2s and T3s-targeted regions in *N. benthamiana*. Thus, some transcription could start within the target region and reduce DNA methylation in wild-type plants, and this reduction might be countered by maintenance mechanisms involving highly accumulated 24-nt siRNAs and/or 25–50-nt RNAs in d2d4 plants. Importantly, demethylases, such as ROS1, might also be involved in the removal of DNA methylation (Figure 9), which is consistent with previous work showing CMV-mediated targeted methylation against a 35S promoter. This methylation was enhanced by down-regulating *ROS1* expression in *N. benthamiana* [41]. Thus, strong maintenance activity of RdDM is required to introduce stable DNA methylation around transcriptional start sites.

The expression of the *NbPDSb*-based inducer efficiently introduced DNA methylation at the homologous sequence of *NbPDSa*, and vice versa (Figure 7A,B and Figure 8A,B). Many small RNAs generated from *NbPDSa-* or *NbPDSb*-based inducers are imperfectly matched with *NbPDSb* or *NbPDSa* sequence, respectively. Thus, perfectly matched siRNAs might direct DNA methylation, and some mismatches might also share this activity. Contrary to our notion that methylation is more effectively induced by perfectly matched inducers than inducers that have mismatches, the expression of NbPDSa-based the T30s inducer introduced DNA methylation in the homologous region of *NbPDSb* more efficiently than NbPDSb-based T3s (Figure 8B). These results suggest that the sequence identity of inducers is not the sole determinant of DNA methylation efficiency. Future detailed analyses of the efficiency of small RNA generation from inducers, sequence composition, and siRNAs responsible for methylation of specific sites will identify factors that determine efficiency and specificity. Further, regardless of similar or higher levels of DNA methylation in *NbPDSb* than in *NbPDSa* (Figure 7B and Figure 8B), the degree of reduction in *NbPDSb* mRNA level was smaller than that for *NbPDSa* mRNA (Figure 7C and Figure 8C). One explanation might be that the degree of reduction was small because of lower basal expression levels of *NbPDSb* (Supplementary Figure S3). Alternatively, regulatory regions required for transcriptional suppression might differ between *NbPDSa* and *NbPDSb*.

In this work, targeted DNA methylation induced by a CMV vector was shown to dynamically change the methylation of an endogenous gene promoter. Additionally, the introduction of DNA methylation against homeologs in *N. benthamiana* was characterized. The findings contribute to the development of efficient targeted DNA methylation and the understanding of epigenetic regulation of polyploid genomes. Targeted DNA methylation by a CMV vector will be a powerful tool to elucidate readily and rapidly functions of specific methylation in a biological context and create plants with altered expression of target genes. The latter development would allow the expression of desirable agronomical traits without changing DNA sequences.

## 4. Materials and Methods

### 4.1. Plant Growth

*N. benthamiana* was grown in soil or hydroponically in a nutrient solution (Otsuka hydroponic composition, Otsuka Chemical Co., Ltd., Osaka, Japan) at 23 °C–25 °C and a 16 h light/8h dark cycle as described earlier [42].

### 4.2. Preparation of Plasmids

For promoter analysis, a genomic fragment of −1225 to −1 from the translational start site of *NbPDSb* was amplified by PCR and exchanged with the 35S promoter upstream of the GUS gene in pBE2113 (pBE2113/NbPDSp::GUS). For targeted DNA methylation with the CMV vector, T2, T3, T30, and asGFP fragments were amplified by PCR and cloned into CMV2 A1 [20] at *Stu*I and *Mlu*I sites using primers described in Supplementary Table S1. The “s” is added as a suffix of each inducer indicates strands coding sense transcripts of the *NbPDS* gene, and “as” is a complementary strand of “s.” For quantification of mRNA levels of *NbPDSa* and *NbPDSb*, fused *NbPDSa* and *NbPDSb* fragments containing amplified region by real-time PCR, and the fragment was cloned into pCR4 (pCR4/NbPDSab-st) according to manufacturer’s instructions. Fragments containing the amplified region of *NbEF1α* by real-time PCR were also cloned into the pCR4 vector (pCR4/NbEF1α-st) as an endogenous reference. Primer information is described in Supplementary Table S1.

### 4.3. Promoter Assay of PDS Gene

*Agrobacterium* cells (LBA4404) transformed with pBE2113/NbPDSp::GUS were suspended in MES buffer (10 mM MES, 10 mM MgCl_2_, pH 5.7), and suspensions were adjusted to an OD 600 nm = 0.5. Acetosyringone was added to the suspensions (final concentration, 150 μM), followed by incubation at room temperature for 2 h. Suspensions were infiltrated into *N. benthamiana* leaves. The infiltrated leaves were immersed in GUS staining buffer (100 mg/mL 5-bromo-4-chloro-3-indolyl-β-D-glucuronide solution was diluted 10 times with 50 mM sodium phosphate buffer, pH = 7.0) and incubated overnight at 37 °C, followed by de-staining with 70% ethanol as described previously [31].

### 4.4. Virus Inoculation

CMV inoculation was conducted following Kanazawa et al. [21]. CMV RNAs 1–3 were obtained by in vitro transcription using linearized plasmids containing cDNAs of RNA1 (pCY1), RNA2 (CMV2 A1 carrying each inducer), or RNA3 (pCY3) with a restriction enzyme. RNAs 1–3 were mixed and rub-inoculated onto *N. benthamiana* leaves.

### 4.5. Bisulfite Sequencing

Genomic DNA was extracted from leaf tissues using the DNeasy Plant Mini Kit (QIAGEN, Hilden, Germany), according to the manufacturer’s instructions. Three hundred ng of genomic DNA was treated using an EZ DNA Methylation-Lightning Kit (ZYMO RESEARCH, Irvin, CA, USA), according to the manufacturer’s instructions. The target region was amplified from the bisulfite-treated genomic DNA using TaKaRa EpiTaq HS polymerase (TaKaRa Bio, Kusatsu, Japan). The reaction mixture (50 μL) contained 1.25 U of TaKaRa EpiTaq HS polymerase, EpiTaq PCR buffer, 3 mM MgCl_2_, 0.2 mM dNTP, 1 μM (each), and forward and reverse primers. Samples were incubated for 10 s at 98 °C, followed by 45 cycles of 98 °C for 10 s, 48 °C for 30 s, and 72 °C for 1 min. Nested PCR was conducted from first-round PCR products using PrimeSTAR GXL DNA Polymerase. The reaction mixture (50 μL) contained 1.25 U of PrimeSTAR GXL DNA polymerase, PrimeSTAR GXL Buffer, 0.2 mM dNTP, 0.6 μM (each) forward and reverse primers. In the second-round PCR, index sequences specific to each sample and linker sequence were added to each primer for deep sequencing. The bulked PCR products from three or four biological replicates were used for each sample. Library preparation and sequencing using MiSeq (Illumina) with 300 bp paired-end were conducted at Hokkaido System Science (Sapporo, Japan) or Bioengineering Lab (Sagamihara, Japan), and methylation was counted using Bismark with the sequence of amplified regions of *NbPDSa* or *NbPDSb* as references. Primer information is described in Supplementary Table S1.

### 4.6. Small RNA Sequencing

The leaf tissues of *N. benthamiana* were homogenized in liquid nitrogen. The total RNA, including small RNAs, was isolated using the mirVana miRNA Isolation Kit according to the manufacturer’s instructions. Library preparation and sequencing were conducted at Hokkaido System Science (Sapporo, Japan) or Bioengineering Lab (Sagamihara, Japan). Bulked total RNAs from three or four biological replicates were used for each sample except for Figure 6, where leaves of a single plant from each line were used. In brief, the library was prepared using a TruSeq Small RNA Library Prep Kit (Illumina) or NEBNext Small RNA Library Prep Set for Illumina (New England Biolabs, Ipswich, MA, USA). Sequencing used either a HiSeq (Illumina) with a 100 bp paired-end or NextSeq (Illumina) with a 75 bp paired-end. Small RNA reads were mapped with a 2-nt mismatch allowed for the *N. benthamiana* Genome v1.0.1 obtained from Sol Genomics Network [32] using Bowtie [43].

### 4.7. Real-Time PCR

The real-time PCR was conducted as described previously [42]. The leaf tissues of *N. benthamiana* were homogenized in liquid nitrogen. The total RNA was isolated using the acid guanidinium thiocyanate–phenol–chloroform (AGPC) extraction method [44], then purified on a FARB minicolumn (Favorgen Biotech Corp., Ping-Tung, Taiwan) [45]. Total RNA was digested with Turbo DNase (Thermo Fisher Scientific, Waltham, MA, USA) and reverse transcribed using random hexamer by PrimeScript II reverse transcriptase (TaKaRa Bio), according to the manufacturer’s instructions. Real-time PCR used the LightCycler 96 system (Roche Diagnostics, Basel, Switzerland). The reaction mixture (10 μL) contained FastStart Essential DNA Probes Master (Roche Diagnostics), 0.5 μM each of forward and reverse primers, 0.2 μM Universal ProbeLibrary Probe (Roche Diagnostics) and cDNA obtained by reverse transcribing 5–10 ng of total RNA. Samples were incubated for 10 min at 95 °C, followed by 45 cycles of 95 °C for 10 s and 60 °C for 30 s. Transcript levels of each gene were normalized to *NbEF1α* (GenBank accession number AY206004). Primers and probes were designed using the Universal ProbeLibrary Assay Design Center (https://qpcr.probefinder.com/organism.jsp, accessed from 2016 to 2018) and are listed in Supplementary Table S2.

Basal expression levels of *NbPDSa* and *NbPDSb* were quantified by real-time PCR using standard plasmids, pCR4/NbPDSab-st for *NbPDSa* and *NbPDSb*, pCR4/NbEF1α-st for *NbEF1α*. *NbPDSa* and *NbPDSb* expression levels were normalized to expression levels of *NbEF1α*.

## 5. Conclusions

In this study, we demonstrated that CMV vector-induced DNA methylation in the promoter region of the *PDS* gene was dynamically changed, and strikingly, the methylation level gradually decreased from the 3′ end of the targeted region, which are blocked by increasing 24-nt siRNAs by down-regulating the *DCL2* and *DCL4* expressions in *N. benthamiana*. We also showed that DNA methylation was induced in the region that contains mismatched sequences to expressed RNAs, suggesting that sequence identity may not be the sole determinant of the efficiency of DNA methylation. These findings contribute to the development of efficient targeted DNA methylation and understanding of epigenetic regulation of polyploid genomes.

## Data Availability

The GenBank/ENA/DDBJ accession numbers for coding sequences of *NbPDSa* (Niben101Scf01283g02002.1) and *NbPDSb* (Niben101Scf14708g00023.1) are LC543532 and LC543533, respectively. Accession numbers for genomic regions of *NbPDSa* (−3260 to +88 from translational start site), *NbPDSb* (−3322 to +94) are LC543534 and LC543535, respectively. Sequencing data have been deposited in the DDBJ Sequenced Read Archive under the accession number DRA010371. These sequences and sequencing data are available at the DNA Data Bank of Japan (DDBJ) (https://www.ddbj.nig.ac.jp/ddbj/index-e.html).

## Supplementary Materials

The following are available online at https://www.mdpi.com/article/10.3390/ijms22084125/s1, Figure S1: Small RNA profiles in wild-type and d2d4 plants infected with CMV vector carrying inducer for DNA methylation; Figure S2: Small RNA profiles in wild-type and d2d4 plants infected with CMV vector carrying inducer for DNA methylation.; Figure S3: Small RNA profiles in wild-type and d2d4 plants infected with CMV vector carrying inducer for DNA methylation.; Figure S4: Sequence alignment of nucleotide sequences of region upstream of translational start.; Figure S5: Time-course analysis of NbPDSa and NbPDSb expressions in plants infected with CMV vector carrying inducer for DNA methylation.; Table S1: Primers used in this study.; Table S2: Primers used in this study.
