# Supplementary material for: Virus-Mediated Targeted DNA Methylation Illuminates the Dynamics of Methylation in an Endogenous Plant Gene
Source: Int J Mol Sci. 2021 Apr 16;22(8):4125. doi: 10.3390/ijms22084125 (PMC8073618; doi:10.3390/ijms22084125)
Supplement: Supplementary file 1 [file ijms-22-04125-s001.zip › ijms-1174164-SI.pdf]

SUPPLEMENTARY MATERIALS

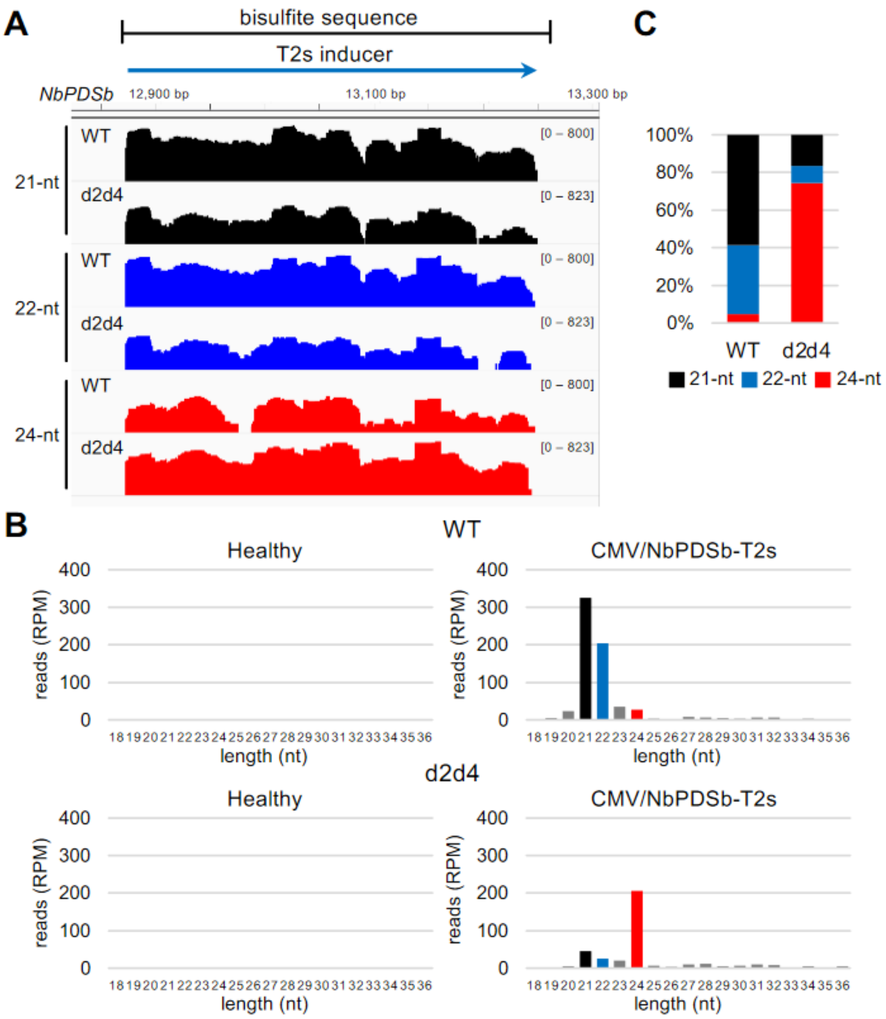

Figure S1. Small RNA profiles in wild-type and d2d4 plants infected with CMV vector carrying inducer for DNA methylation (supports Figure 5). Small RNA sequencing used total RNAs from the leaves where T2s inducer (−938 to −568) was expressed by the CMV vector at 21 days after inoculation. **(A)** IGV snapshots of 21-nt, 22-nt, and 24-nt small RNAs in the region analyzed by bisulfite sequencing (−957 to −565). The *y* axes indicate raw reads on a log scale and were normalized by total mapped reads. **(B)** Length distribution of 18–36-nt small RNAs.**(C)** Ratios of 21-nt, 22-nt, 24-nt small RNAs.

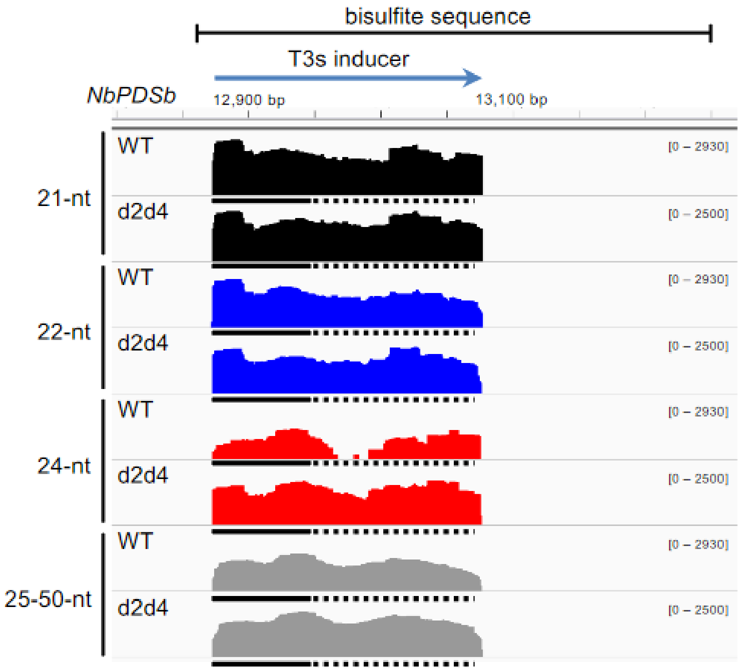

Figure S2. Small RNA profiles in plants infected with CMV carrying T3s inducer at 35 days after inoculation (supports Figure 5). Small RNA sequencing used total RNAs from the leaves where T3s inducer (−938 to −739) was expressed by the CMV vector at 35 days after inoculation. IGV snapshots of 21-nt, 22-nt, 24-nt, 20–50-nt small RNAs in the regions analyzed by bisulfite sequencing (−957 to −565) in **Figure 5A**. The *y* axes indicate raw reads on a log scale and were normalized by total mapped reads. Black solid and broken lines indicate high and low methylation regions in wild-type relative to d2d4 plants at 35dpi, respectively as shown in **Figure 5A**.

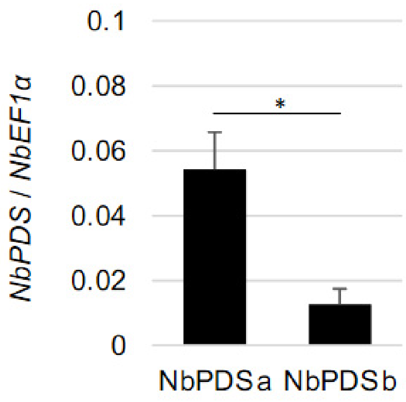

Figure S3. Basal expression levels of *NbPDSa* and *NbPDSb* in *N. benthamiana* (supports Figures 7 and 8). *NbPDSa* and *NbPDSb* expressions were analyzed by real-time PCR. The expression levels of *NbPDSa*, *NbPDSb* and *NbEF1α* were calculated using standard plasmids containing target regions and were normalized relative to *NbEF1α*. Error bars indicate

standard deviation of four biological replicates. Statistical analyses were conducted using Welch's *t* test. *, $P < 0.05$.

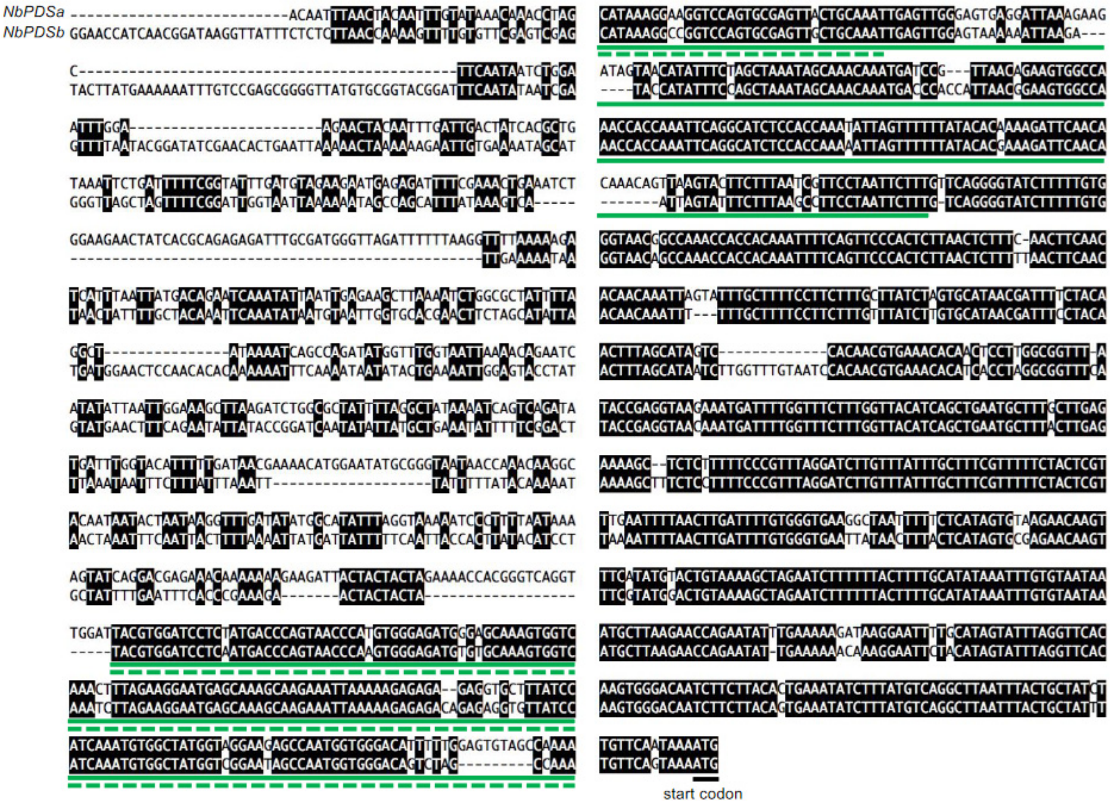

Figure S4. Sequence alignment of nucleotide sequences of region upstream of translational start (supports Figures 7 and 8). Genomic DNA sequence of *NbPDSa* (−1500 to +3 from translational start site) and *NbPDSb* (−1500 to +3) were aligned by the program Pro-Coffee (http://tcoffee.vital-it.ch/apps/tcoffee/do:procoffee). Green solid line indicates T2 region. Green broken line indicates T3 and T30 regions.

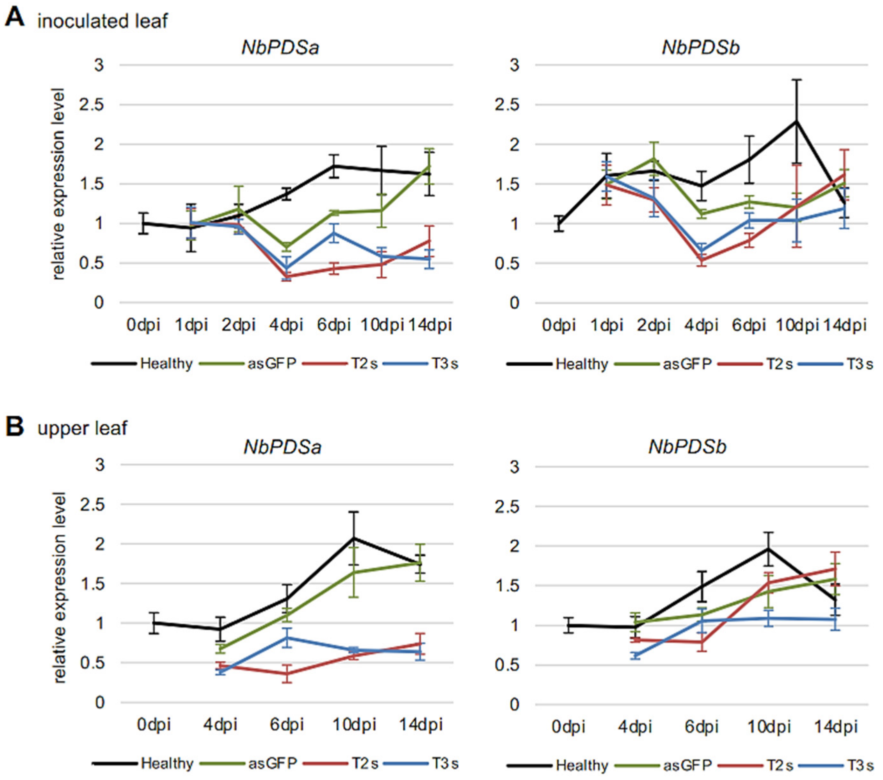

Figure S5. Time-course analysis of *NbPDSa* and *NbPDSb* expressions in plants infected with CMV vector carrying inducer for DNA methylation (supports Figure 7). **(A)** and **(B)** mRNA levels of *NbPDSa* and *NbPDSb* were separately quantified by real-time PCR in the inoculated **(A)** and upper non-inoculated leaves **(B)** with CMV/NbPDSb-T2s, -T3s, -asGFP (vector control). Expression level at 0 dpi was set to 1.0 for each *NbPDSa* and *NbPDSb*. The expression levels were normalized relative to that of *NbEF1α*. Error bars indicate standard deviation of four biological replicates.

Table S1. Primers used in this study.

Experiment		primer		
			#	sequence (5'-3')
construction of CMV vector	pCY2/NbPDSb-T2s	Fw	202	actaggcctgaTACGTGGATCCTCAATGACC
		Rv	199	actacgcgtAAAGAATTAGGAAGGCTTAAAG
	pCY2/NbPDSb-T3s	Fw	202	actaggcctgaTACGTGGATCCTCAATGACC
		Rv	204	actacgcgtATTTGCAGCAACTCGCACTG
	pCY2/NbPDSb-T2as	Fw	201	actaggcctgaAAAGAATTAGGAAGGCTTAAAG
		Rv	203	actacgcgtTACGTGGATCCTCAATGACC
	pCY2/NbPDSb-T3as	Fw	205	actaggcctgaATTTGCAGCAACTCGCACTG
		Rv	203	actacgcgtTACGTGGATCCTCAATGACC
	pCY2/NbPDSb-T30s	Fw	883	actaggcctgaTACGTGGATCCTCTATGACCCA
		Rv	884	actacgcgtATTTGCAGTAACTCGCACTG
	pCY2/asGFP	Fw	156	actaggcctgaCGTCCTCCTTGAAATCGATTC
		Rv	157	actacgcgtATGAGTAAAGGAGAAGAACT
construction of binary vector	pBE2113/NbPDSp::GUS	Fw	PDSp_F	tagttaGATATCTGTAATTGGTGCACGAACTTCTAGC
		Rv	PDSp_R	tagttaTCTAGATTTACTGAACAAAATAGCAGTAAATTAAGCCTG
template for real-time PCR	pCR4/NbPDSa+NbPDSb	Fw	1005	TGCTGGAGGCAAGAGATGTC
		Rv	1011	ttggactctcaaattaacggTGACTTTCTCGGGCCACGTA
		Fw	1012	TACGTGGCCCGAGAAAGTCAccgttaatttgagagtccaa
		Rv	1010	TTGTTGGGCGTGAGGAAGTAC
	pCR4/NbEF1α	Fw	1090	GCTGAACGTGAGCGTGGTATCAC
		Rv	1091	CATACCAGGCTTGAGGACAC
bisulfite sequene	NbPDSa-1st PCR	Fw	1039	GGTTTGATATATGGyATATTTAGG
		Rv	1040	CAAArAArrAAAArCAAATAC
	NbPDSa-2nd PCR	Fw	1042	AATAAAAGTATyAGGAyGAG
		Rv	327	TTACCCACAAAAArATACCCCT
	NbPDSb-1st PCR	Fw	325	AyATyyTGyTATTTTGAATTT
		Rv	329	AAATCrTTATrCACAArATAAAC
	NbPDSb-2nd PCR	Fw	324	ATyyTGyTATTTTGAATTTyA
		Rv	327	TTACCCACAAAAArATACCCCT

Table S2. Primers and probes used for real-time PCR.

gene	primer			probe#*
		#	sequence (5'-3')	
NbPDSa+NbPDSb	Fw	742	TGCAGAACCTGTTTGGAGAA	86
	Rv	743	GCTTGTTAGGCATCGCAAAT	
NbPDSa	Fw	828	TTGGAGAACTAGGGATTGAT	86
	Rv	829	GTTAGGCATCGCAAATATCATT	
NbPDSb	Fw	784	TCCATGGGGCACAAGTTTAG	118
	Rv	785	TCCTTTGTCAATCTTCTGGTCA	
CMV	Fw	500	AATGAAGAGTGCTCATTTGACG	55
	Rv	501	AACGTATGCAGCGGATGAT	
NbEF1α	Fw	179	CTGGTACCTCCCAAGCTGAC	56
	Rv	180	CCAGCTTCAAAACCACCAGT	

*UPL universal probe library (Roche Diagnostics).
